# Dietary and Sedentary Behavior in Children and Adolescents

**DOI:** 10.3390/nu17071178

**Published:** 2025-03-28

**Authors:** Rubén Trigueros, Jose M. Aguilar-Parra

**Affiliations:** Hum-878 Research Team, Health Research Centre, Department of Psychology, University of Almería, 04120 Almería, Spain; jmaguilar@ual.es

## 1. Introduction

Childhood and adolescence are critical stages for the development of lifelong health habits that shape one’s overall well-being and risk of developing diseases later in life [1]. During these formative years, dietary behaviors and physical activity levels are established, influencing both physical and mental health outcomes [2]. As such, understanding the factors that impact children’s and adolescents’ eating patterns and lifestyle choices is essential for facilitating the design of effective public health interventions that promote healthy development [1,3].

In recent decades, changes in global food systems, economic structures, technological advancements, and sociocultural norms have contributed to shifts in dietary behaviors and a trend towards sedentary lifestyles among younger populations [4,5]. The increased consumption of ultra-processed foods, a growing reliance on convenience meals, and the declining physical activity levels in these populations have raised concerns among public health professionals, educators, and policymakers [5], as the consequences of these lifestyle changes are evident in the rising rates of childhood obesity, metabolic disorders, and mental health challenges [6].

One of the most pressing dietary concerns in children and adolescents is the excessive intake of processed foods, which are often high in refined sugars, unhealthy fats, and sodium, while lacking in essential nutrients such as fiber, vitamins, and minerals [7,8]. These dietary patterns have been linked to an increased risk of obesity, type 2 diabetes, cardiovascular diseases, and other non-communicable conditions [9,10]. Moreover, disparities in food accessibility, influenced by one’s socio-economic status and geographic location, further complicate efforts to ensure that all children have access to a balanced diet [11,12].

The family environment plays a pivotal role in shaping dietary habits from an early age. Parents and caregivers influence their children’s food preferences, meal structures, and eating behaviors through the availability of food in the home, their meal preparation practices, and the degree to which they model healthy eating behaviors [13]. However, external factors such as advertising, the influence of peers, and school meal offerings also contribute significantly to children’s dietary choices [14]. Digital media and social networking platforms have further complicated this landscape, as exposure to food marketing and influencer-promoted eating trends can shape children’s perceptions of what constitutes a healthy diet [15].

In addition to dietary concerns, the rise in sedentary behavior among children and adolescents poses a major public health challenge [1]. With increasing screen time among younger populations due to digital entertainment, online learning, and social media engagement, physical activity levels have declined. The COVID-19 pandemic exacerbated these trends, as lockdowns and school closures resulted in further reductions in opportunities to engage in physical activity [16]. This is cause for concern, as studies have shown that prolonged screen time is associated with negative health outcomes such as poor metabolic health, disrupted sleep patterns, increased anxiety, and reduced cognitive function.

The intersection between the effects of dietary behaviors and sedentary lifestyles underscores the need for comprehensive interventions that promote healthy eating and active living among children and adolescents [17]. Schools, families, and communities must work together to create environments that support nutritious food choices and engaging in regular physical activity. Initiatives such as nutrition-related education programs, regulations on school meals, and community-based fitness opportunities can play a crucial role in reversing current trends and improving long-term health outcomes for young people [18].

This Special Issue explores the various dimensions of dietary and sedentary behavior in children and adolescents, presenting the findings from a number of past studies that have examined the key determinants of health-related behaviors, the impact of family and school environments, and the effectiveness of intervention programs. By identifying the factors that shape children’s and adolescents’ lifestyle choices, these studies provide valuable insights for the development of evidence-based strategies to promote lifelong well-being.

## 2. Dietary Behavior in Children and Adolescents

The dietary behavior of children and adolescents is affected by multiple factors, including food availability and accessibility, the influence of family and school, food industry marketing strategies, and the impact of social networks [1,19]. In many countries, there has been an increase in the consumption of ultra-processed foods and sugary drinks, accompanied by a reduction in the intake of fruits, vegetables, and other foods rich in essential nutrients [6]. These dietary trends may contribute to the development of nutritional imbalances and an increased risk of chronic diseases later in life [2].

The quality of children’s diets is determined by the availability of healthy food at home and its accessibility in schools [1,6]. Families with a lower socio-economic status may face difficulties in accessing fresh and nutritious food, relying more heavily on processed and cheap products with lower nutritional value [5,20]. In addition, advertising and marketing aimed at children have been shown to significantly influence their food preferences, promoting the consumption of products which are high in sugars and saturated fats [4].

Social media and digital platforms have also emerged as key factors shaping eating habits [1]. The exposure to content sponsored by influencers and brands can generate misperceptions about healthy eating in children, driving the popularization of extreme diets or the consumption of products with low nutritional value [1]. It is crucial that intervention strategies consider these cultural aspects in order to achieve sustainable changes in children’s and adolescents’ diets [6].

## 3. Sedentary Behavior and Its Implications for Children’s Health

In parallel, sedentary behavior, characterized by engagement in low-energy-expenditure activities such as those involving screen time, has increased significantly among children and adolescents [3]. Reduced physical activity and increased sedentary behavior are associated with negative effects on metabolic health, cognitive function, and emotional well-being [1]. The COVID-19 pandemic exacerbated these trends, with an increase in the time spent engaging in sedentary activities due to mobility restrictions and school closures [5,21].

The excessive use of electronic devices and the scarcity of adequate spaces for physical activity have reduced opportunities for children to exercise and engage in active play [2]. Studies have shown that prolonged screen time may be linked to increased childhood obesity, poorer sleep quality, and mental health problems such as increased stress and anxiety [6].

The impact of sedentary lifestyles on cognitive development is another cause for concern. It has been shown that children with higher levels of physical activity tend to have better academic performance and a greater ability to concentrate [4]. This underlines the importance of policies that promote movement within the school and community environment [2]. In addition, well-structured physical education programs which are accessible to all children can play an essential role in reducing their time spent engaging in sedentary activities.

## 4. Main Results of the Previous Studies

- Determinants of Diet Quality in Adolescents: Results from the Prospective Population-Based EVA-Tyrol and EVA4YOU Cohorts [2]. This study investigated diet quality and its determinants in adolescents from two European cohorts. The results showed that only 8% of the adolescents met the recommended vegetable intake, while a significant proportion exceeded the recommended intakes of sodium and red and processed meats. A better diet quality was found to be associated with being female, engaging in regular physical activity, having attained a higher level of education, and possessing greater nutritional knowledge. These findings highlight the need for targeted interventions to improve the quality of adolescents’ diets.
- Impact of the Family Environment on the Frequency of Animal-Based Product Consumption in School-Aged Children in Central Poland [4]. While the specific details of this study were not found in the available sources, similar research has shown that the family environment significantly influences children’s eating habits, with one such factor including the frequency of animal-based product consumption.
- Ultra-Processed Food vs. Fruit and Vegetable Consumption Before and During the COVID-19 Pandemic Among Greek and Swedish Students [5]. No specific details were found regarding this study in the available sources; however, previous research has indicated that the COVID-19 pandemic affected students’ eating habits, leading to an increase in their ultra-processed food consumption and a decrease in their fruit and vegetable intake.
- Pilot Study on Satisfaction in Children and Adolescents After a Comprehensive Educational Program on Healthy Habits [6]. This study evaluated the “MotivACTION” educational program, which was designed to promote healthy habits among children and adolescents. The participants, aged 8 to 14, reported their high level of satisfaction with the program. Additionally, as a result of the program, they demonstrated the ability to create healthy menus, indicating a practical understanding of the concepts that they had been taught.
- Intuitive Eating Behaviour Among Young Malay Adults in Malaysian Higher Learning Institutions [3]. This study examined the intuitive eating behaviors of 367 young Malay adults. The findings indicated that greater engagement in intuitive eating was associated with a lower prevalence of obesity, fewer restrictive diets, healthier weight control behaviors, and fewer binge-eating episodes. Gender differences were identified in the responses to certain intuitive eating subscales, and there was a significant relationship between weight control behaviors, binge-eating episodes, and dieting.
- A Systematic Review of Healthy Nutrition Intervention Programs in Kindergarten and Primary Education [1]. While the specific details of this review were not found in the available sources, similar studies have emphasized the effectiveness of early nutrition intervention programs in promoting healthy eating habits and preventing childhood obesity.

## 5. Conclusions and Future Perspectives

It is essential that public policies comprehensively address the promotion of healthy habits, ensuring equitable access to nutritious food and fostering environments that promote physical activity. The results of the studies included in this Special Issue reinforce the importance of multidimensional interventions that consider both the socio-economic context and the impact of technology on children’s lifestyles.

The future of children’s health depends on society’s ability to respond to these challenges with innovative and sustainable solutions. The implementation of educational programs from early childhood, which promote a balanced diet and reduced sedentary time, can make a significant difference to the quality of life of future generations.

In addition, it is essential to encourage families to become increasingly involved in creating healthy food environments at home and to promote equitable access to sports and recreational facilities, thereby ensuring that all children have the opportunity to be physically active.

Educational institutions, as spaces where children and adolescents spend much of their time, therefore play a crucial role in this task. Initiatives such as the inclusion of nutrition education in school curricula, the regulation of food offerings in cafeterias, and the promotion of active recess can contribute significantly to improving the health of young people.

Collaboration between governments, researchers, health professionals, and the food industry will be key to developing effective and sustainable strategies to improve children’s health. Policies should be designed to regulate the advertisement of unhealthy foods to children, while encouraging the consumption of more nutritious options.

In conclusion, this Special Issue highlights the importance of continuing to research and implement evidence-based strategies to address the challenges associated with poor diets and sedentary lifestyles in childhood and adolescence. Joint action across multiple sectors will enable progress towards a healthier society, where future generations can grow up in an environment that supports their physical, mental, and emotional well-being.
